# Randomized phase-II trial evaluating induction therapy with idarubicin and etoposide plus sequential or concurrent azacitidine and maintenance therapy with azacitidine

**DOI:** 10.1038/s41375-019-0395-y

**Published:** 2019-02-06

**Authors:** R. F. Schlenk, D. Weber, W. Herr, G. Wulf, H. R. Salih, H. G. Derigs, A. Kuendgen, M. Ringhoffer, B. Hertenstein, U. M. Martens, M. Grießhammer, H. Bernhard, J. Krauter, M. Girschikofsky, D. Wolf, E. Lange, J. Westermann, E. Koller, S. Kremers, M. Wattad, M. Heuser, F. Thol, G. Göhring, D. Haase, V. Teleanu, V. Gaidzik, A. Benner, K. Döhner, A. Ganser, P. Paschka, H. Döhner

**Affiliations:** 1 Department of Internal Medicine III, University Hospital of Ulm, Ulm, Germany; 2 NCT-Trial Center, National Center of Tumor Diseases, Heidelberg University Hospital and German Cancer Research Center, Heidelberg, Germany; 3 Department of Internal Medicine V, Heidelberg University Hospital, Heidelberg, Germany; 4 Department of Hematology, Medical Oncology and Pneumology, University Medical Center Mainz, Mainz, Germany; 5 Department of Hematology and Oncology, University Hospital of Göttingen, Göttingen, Germany; 6 Department of Hematology and Oncology, Eberhard-Karls University, Tübingen, Germany; 7 Department of Internal Medicine III, Hospital Frankfurt-Hoechst, Frankfurt, Germany; 8 Department of Hematology, Oncology and Clinical Immunology, University of Duesseldorf, Medical Faculty, Duesseldorf, Germany; 9 Department of Hematology and Oncology, Städtisches Klinikum Karlsruhe, Karlsruhe, Germany; 10 Department of Hematology and Oncology, Klinikum Bremen Mitte, Bremen, Germany; 11 Department of Hematology and Oncology, Klinikum am Gesundbrunnen, Heilbronn, Germany; 12 Department of Hematology and Oncology, University Hospital of Minden, Minden, Germany; 13 Department of Hematology and Oncology, Darmstadt, Municipal Hospital, Darmstadt, Germany; 14 Department Hematology and Oncology, Braunschweig Municipal Hospital, Braunschweig, Germany; 15 Department of Hematology and Oncology, Hospital Elisabethinen Linz, Linz, Austria; 16 Internal Medicine III, University Hospital of Bonn, Bonn, Germany; 17 Department of Internal Medicine V, Medical University Innsbruck, Innsbruck, Austria; 18 Department of Hematology and Oncology, Evangelisches Krankenhaus Hamm, Hamm, Germany; 19 Department of Hematology, Oncology and Tumor Immunology, Charité - Campus Virchow Clinic, Berlin, Germany; 20 Department of Internal Medicine III, Hanuschkrankenhaus Wien, Wien, Austria; 21 Department of Internal Medicine, Caritas-Krankenhaus Lebach, Lebach, Germany; 22 Department of Hematology and Oncology, Hospital Essen-Werden, Essen, Germany; 23 Department of Hematology, Hemostasis, Oncology, and Stem Cell Transplantation, Hannover Medical School, Hannover, Germany; 24 Institute of Human Genetics, Hannover Medical School, Hannover, Germany; 25 Division of Biostatistics, German Cancer Research Center Heidelberg, Heidelberg, Germany; 26 Present Address: Department of Hematology and Internal Oncology, University Hospital of Regensburg, Regensburg, Germany

**Keywords:** Drug development, Randomized controlled trials

## Abstract

The aim of this randomized phase-II study was to evaluate the effect of substituting cytarabine by azacitidine in intensive induction therapy of patients with acute myeloid leukemia (AML). Patients were randomized to four induction schedules for two cycles: STANDARD (idarubicin, cytarabine, etoposide); and azacitidine given prior (PRIOR), concurrently (CONCURRENT), or after (AFTER) therapy with idarubicin and etoposide. Consolidation therapy consisted of allogeneic hematopoietic-cell transplantation or three courses of high-dose cytarabine followed by 2-year maintenance therapy with azacitidine in the azacitidine-arms. AML with *CBFB*-*MYH11*, *RUNX1*-*RUNX1T1*, mutated *NPM1*, and *FLT3*-ITD were excluded and accrued to genotype-specific trials. The primary end point was response to induction therapy. The statistical design was based on an optimal two-stage design applied for each arm separately. During the first stage, 104 patients (median age 62.6, range 18–82 years) were randomized; the study arms PRIOR and CONCURRENT were terminated early due to inefficacy. After randomization of 268 patients, all azacitidine-containing arms showed inferior response rates compared to STANDARD. Event-free and overall survival were significantly inferior in the azacitidine-containing arms compared to the standard arm (*p* < 0.001 and *p* = 0.03, respectively). The data from this trial do not support the substitution of cytarabine by azacitidine in intensive induction therapy.

## Introduction

Acute myeloid leukemia (AML) is predominantly a disease of older patients for whom prognosis remains poor [1, 2]. Intensive chemotherapy, usually consisting of an anthracycline and cytarabine, induces remission in about 50% of older fit patients, but most patients relapse and succumb to their disease. Beyond patient-associated factors, such as increasing age, comorbidities and poor performance status, disease-related factors and particularly an unfavorable genetic profile of the disease predicts resistance to current standard therapy [3, 4]. In line, the proportion of patients with an unfavorable disease profile such as intermediate-2 and high risk according to 2010 European LeukemiaNet (ELN) [5] recommendations increases with older age from about one third in patients below the age of 60 years to nearly 60% in patients 70 years or older [6].

Epigenetic changes, such as mutations of epigenetic modifiers and aberrant DNA methylation, are frequent in AML [7, 8]. Furthermore, DNA methylation has emerged as an attractive therapy target in AML [9, 10] and particularly in patients with unfavorable cytogenetic and/or a *TP53* mutation [11, 12]. The high failure rate of intensive induction therapy in AML with an unfavorable genetic profile may be a result of cytarabine resistance [13]. In contrast, patients with a favorable genetic profile such as core-binding factor (CBF) AML or AML with mutated *NPM1* are sensitive to standard induction therapy [3, 4]. The improved understanding of the molecular pathogenesis has spurred new treatment strategies targeting specific molecular defects. So far, this concept has been successful by using all-trans retinoic acid (ATRA) and/or arsenic trioxide in the therapy of acute promyelocytic leukemia (APL) [14], as well as by introducing midostaurin and enasidenib in the therapy of AML with *FLT3* and *IDH2* mutations, respectively [15, 16].

In this trial, patients with CBF-AML [17], AML with mutated *NPM1* [18], and AML with *FLT3* internal tandem duplication (ITD) [19] were excluded due to competitive trials that were active during the same time resulting in a selection of patients with more high-risk disease features. The hypothesis was that these patients may particularly benefit from incorporation of the hypomethylating agent azacitidine in induction therapy. Thus, the aim of our study was to evaluate the impact of substituting cytarabine by azacitidine administered sequentially or concurrently with idarubicin and etoposide on response rate and survival endpoints. The AMLSG 12–09 trial was a prospective, randomized, multi-institutional, controlled phase-II trial.

## Patients and methods

### Patients

Between October 2010 and March 2012, 277 adult patients 18–82 years of age with newly diagnosed AML were enrolled; diagnoses included de novo AML, secondary AML with a preceding history of myelodysplastic syndrome or myeloproliferative neoplasm (s-AML), and therapy-related AML following treatment of a primary malignancy (t-AML), as defined by the WHO 2008 classification [20]. Excluded were APL, CBF-AML, AML with *FLT3*-ITD, and AML with *NPM1* mutation; further exclusion criteria were concomitant renal (creatinine > 1.5 x upper normal serum level), liver (AST or ALP > 2.5 x upper normal serum level) or cardiac dysfunction (New York Heart Association III/IV), uncontrolled infectious disease, primary coagulation disturbance or ECOG performance status > 2. Written informed consent was obtained from all patients. The protocol was approved by the lead Ethics Committee and registered at clinicaltrialsregister.eu (EudraCT Number: 2009-016142-44) and clinicaltrials.gov (ClinicalTrials.gov Identifier: NCT01180322).

### Cyto- and molecular genetics

Chromosome banding analysis was performed centrally in the two AMLSG Laboratories for Cytogenetics (Hannover, Ulm). Karyotypes were designated according to the International System for Human Cytogenetic Nomenclature [21]. Leukemia samples were analyzed for mutations in *FLT3* (ITDs and tyrosine kinase domain [TKD] mutations at codons D835/I836), *CEBPA*, *NPM1*, *IDH1/2*, *RUNX1, ASXL1, TP53*, and *DNMT3A* as previously described [22–27].

### Study design

Patients were randomized into 4 arms in a 1:1:1:1 manner. Induction therapy regimens comprised: a) STANDARD, cytarabine 100 mg/m²/day by continuous intravenous (iv) infusion on days 1–7, idarubicin 12 mg/m²/day by iv push on days 1,3,5 (application in patients > 65 years at days 1 + 3 only), etoposide 100 mg/m²/day by 1-hour iv infusion on days 1,2,3 (application in patients > 65 years at days 1 + 3 only); b) Azacitidine PRIOR, azacitidine 100 mg/m²/day by subcutaneous (sc) injection on days -5 to day −1, idarubicin and etoposide as in STANDARD; c) Azacitidine CONCURRENT, azacitidine 100 mg/m²/day by sc injection on days 1–5 concurrently to idarubicin and etoposide as in STANDARD; d) Azacitidine AFTER, azacitidine 100 mg/m² per day by sc injection on days 4–8. Patients in complete remission (CR), CR with incomplete hematological recovery (CRi) or partial remission (PR) after first induction therapy received a second cycle with a dose reduction of idarubicin (administered on days 1 + 3 only).

#### Consolidation therapy

Patients in CR/CRi following induction therapy were assigned to consolidation therapy with either allogeneic hematopoietic-cell transplantation (HCT) from a matched related or unrelated donor (one consolidation cycle before allogeneic HCT was optional), or, in second priority, three cycles of high-dose cytarabine (HiDAC). Cytarabine was administered by iv infusion in a dose of 3 g/m² bid on days 1,2,3 [28]. For patients > 65 years of age, dose of cytarabine was reduced to 1 g/m². Lenograstim (34 × 10^6^IU/ml) was applied sc daily beginning on day 10 until neutrophil count > 0.5 × 10^9^/l.

#### Maintenance therapy

with azacitidine was intended in all patients who were randomized to one of the azacitidine-containing induction therapy arms. Maintenance therapy was scheduled for a total duration of 2 years. Azacitidine was administered in a dose of 50 mg/m² per day by sc injection on days 1–5 every 4 weeks.

### Definition of response criteria, survival endpoints and hematologic recovery

In accordance with standard criteria, CR was defined as < 5 % bone marrow blasts, an absolute neutrophil count of ≥ 1.0 G/L, a platelet count of ≥ 100 G/L, no blasts in the peripheral blood and no extramedullary leukemia; CR with incomplete blood count recovery (CRi) was characterized as CR except for residual neutropenia (neutrophils < 1.0 G/L) or thrombocytopenia (platelets < 100 G/L) [5]. Relapse was defined as > 5% bone marrow blasts or new extramedullary leukemia in patients with previously documented CR/CRi.

Event-free survival (EFS), relapse-free survival (RFS) and overall survival (OS) were defined as recommended [5]. Times to leukocyte, neutrophil and platelet recovery were measured from the first day of chemotherapy of each cycle until the first day with values ≥ 1, ≥ 0.5 and ≥ 20 G/L for white blood cells (WBC), neutrophils and platelets, respectively. Toxicities were defined and graded according to the National Cancer Institute (NCI) Common Toxicity Criteria, version 3.0.

### Sample size planning and statistical analysis

An optimal two-stage design of Simon was used to evaluate each arm of the study separately [29]. The null hypothesis in each arm was H_0_: *π* ≤ 0.40, whereby *π* denoted the true CR/CRi rate of the induction therapy. In contrast, an effective therapy was estimated to achieve at least a CR/CRi rate of 55%. The sample size was calculated to detect an effective therapy with a power of 80%. The level of significance was fixed at *α* = 5% for each treatment arm. Based on the assumptions an efficacy of the corresponding therapy was rejected in the first stage of 26 treated patients, if 11 or less patients achieved a CR/CRi. If 12 or more patients achieved a CR/CRi during this first stage, the trial proceeded to second stage with a total sample size of 84 patients per treatment arm. Randomization after completion of the first stage was carried on until first stage results were available. Second stage rejection was considered if not more than 40 patients achieved a CR/CRi.

Pairwise comparisons between patient subgroups were performed by the Mann–Whitney or Kruskal-Wallis test for continuous variables and by Fisher’s exact test for categorical variables. Univariable and multivariable logistic regression models were applied to investigate the influence of covariates (age, sex, *CEBPA*, *DNMT3A, RUNX1, ASXL1, IDH1, IDH2*, *TP53*, ELN high-risk category) on response to induction therapy. Secondary endpoints of the study were OS, RFS, EFS, therapy-related toxicity and their correlation with the study drug. The median duration of follow-up was calculated by the reverse Kaplan–Meier estimate [30]; the Kaplan–Meier method was used to estimate the distributions of EFS, RFS and OS. Survival distributions were compared using the log-rank test. Multivariable Andersen-Gill regression models were used to evaluate the same prognostic variables as for response to induction therapy as well as alloHCT as a time-dependent covariable [31]. Missing data were replaced by 50 imputations using multivariate imputations by chained equations applying predictive mean matching [32]. Backward selection applying a stopping rule based on a p-value of 0.50 was used in multivariable regression models to exclude redundant or unnecessary variables [32].

All statistical analyses were performed with the statistical software environment R, version 3.2.1, using the R packages rms, version 4.3-1, and cmprsk, version 2.2-2 [33].

## Results

### Patients and baseline characteristics

**Fig. 1:**
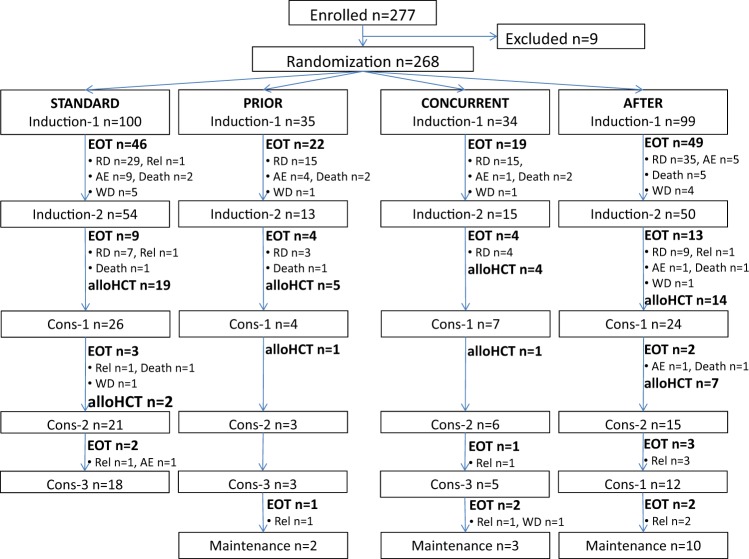
CONSORT Diagram. Abbreviations: STANDARD, Cytarabine 100 mg/m²/day by continuous iv infusion on days 1–7, idarubicin 12 mg/m²/day by iv push on days 1,3,5 (application in patients > 65 years at days 1 + 3 only), etoposide 100 mg/m²/day by 1-hour iv infusion on days 1,2,3 (application in patients > 65 yrs at days 1 + 3 only); PRIOR, Azacitidine 100 mg/m² per day by subcutaneous injection on days -5 to day −1, idarubicin and etoposide as in STANDARD; CONCURRENT, Azacitidine 100 mg/m²/day by sc injection on days 1–5, idarubicin and etoposide as in STANDARD; AFTER, Azacitidine 100 mg/m² per day by sc injection days 4–8, idarubicin and etoposide as in STANDARD; EOT, end of trial; RD, refractory disease; Rel, relapse; AE, adverse event; WD, withdrawal; alloHCT, allogeneic hematopoietic-cell transplantation; Cons, consolidation

Of 277 patients, 9 (3%) were excluded due to violation of inclusion/exclusion criteria: no diagnosis of AML, *n* = 3; AML with *NPM1* mutation, *n* = 1; presence of Philadelphia chromosome, *n* = 1; organ insufficiency (renal failure), *n* = 1; withdrawal of informed consent, *n* = 2; other reason (extramedullary manifestation of AML in spleen), *n* = 1. Thus, overall 268 patients were randomized (Fig. 1).

On the basis of the two-stage design of the study, initially 104 patients were randomized in the first stage between October 2010 and September 2011 with equal distribution into the 4 arms (*n* = 26 each). The baseline characteristics of all randomized patients as well as those randomized during the first stage (data not shown) were equally distributed except for the frequency of inv(3)/t(3;3) (Table 1).

**Table 1:** Patient and disease characteristics according to randomization

	All patients (*n* = 268)	STANDARD (*n* = 100)	PRIOR (*n* = 35)	CONCURRENT (*n* = 34)	AFTER (*n* = 99)	*p* value
Age in years Median (Range)	62.6 (18–83)	62.6 (20–81)	63.5 (21–79))	63.6 (36–83)	62.3 (18–79)	0.73
ECOG Performance Status						
0, No. (%)	113 (42)	42 (42)	16 (46)	13 (38)	42 (42)	0.94
1–2, No. (%)	155 (58)	58 (58)	19 (54)	21 (62)	57 (58)	
Male sex, No. (%)	144 (54)	49 (49)	21 (60)	14 (41)	60 (61)	0.14
Missing	6	2	0	2	2	
WBC, 10^9^/L (*n* = 262)						
Median (Range)	3.7 (0.3–214)	3.0 (0.4–186)	3.4 (0.8–155)	3.6 (0.6–141)	4.2 (0.3–214)	0.78
Hemoglobin, g/dL (*n* = 262)						
Median (Range)	9.2 (5.2–13.8)	9.1 (5.2–12.9)	9.8 (5.9–12.9)	9.6 (6.9–13.6)	9.1 (5.6–13.8)	0.33
Platelets, 10^9^/L (*n* = 262)						
Median (Range)	63.5 (3–1286)	64.5 (3–419)	61 (16–1286)	53 (12–301)	67 (4–956)	0.77
^a^Bone marrow blasts, % (*n* = 252)						
Median (Range)	60 (0–100)	53 (0–100)	63.5 (10–100)	61.5 (20–93)	60 (0–99)	0.84
Peripheral blood blasts, % (*n* = 246)^a^						
Median (Range)	10 (0–97)	12 (0–97)	16 (0–88)	5 (0–92)	8.5 (0–97)9	0.95
Cytogenetics (*n* = 230)						
Normal karyotype, *n* (%)	93 (40)	38 (45)	10 (30)	11 (44)	34 (39)	0.69
inv(3)/t(3;3), *n* (%)	5 (2)	2 (2)	3 (9)	0 (0)	0 (0)	0.02
t(11q23;var), *n* (%)	11 (5)	6 (7)	1 (3)	1 (4)	3 (3)	0.79
Myelodysplasia-related karyotype, n (%)^b^	72 (33)	27 (34)	10 (32)	5 (21)	30 (35)	0.55
Complex, *n* (%)^b^	46 (20)	15 (18)	5 (15)	5 20)	21 (24)	0.61
Monosomal, *n* (%)^b^	32 (15)	10 (13)	4 (13)	2 (8)	16 (19)	0.69
AML type (*n* = 268)						
de novo AML, *n* (%)	197 (74)	73 (73)	24 (69)	24 (71)	76 (77)	0.68
s-AML, *n* (%)	50 (19)	16 (16)	8 (23)	8 (24)	18 (18)	
t-AML, *n* (%)	21 (8)	11 (11)	3 (9)	2 (6)	5 (5)	
Mutated *CEBPA* (*n* = 238)						
Single mutant n (%)	6 (3)	4 (4)	0 (0)	0 (0)	2 (2)	0.81
Double mutant, n (%)	17 (7)	7 (8)	1 (3)	1 (3)	8 (9)	
Mutated *DNMT3A*, *n* (%) (*n* = 235**)**	40 (17)	17 (18)	6 (21)	6 (20)	11 (13)	0.66
Mutated *ASXL1*, *n* (%) (*n* = 213)	32 (15)	16 (20)	5 (19)	2 (7)	9 (12)	0.34
Mutated *RUNX1*, *n* (%) (*n* = 233)	46 (20)	15 (16)	8 (30)	9 (30)	14 (17)	0.17
Mutated *IDH1*, *n* (%) (*n* = 231)	25 (11)	13 (14)	3 (11)	3 (10)	6 (7)	0.53
Mutated *IDH2*, *n* (%) (*n* = 233)	32 (14)	16 (17)	5 (19)	4 (13)	7 (8)	0.23
Mutated *TP53*, *n* (%) (*n* = 215)	27 (13)	5 (6)	3 (11)	6 (21)	13 (17)	0.08

*STANDARD* cytarabine 100 mg/m²/day by continuous iv infusion on days 1–7, idarubicin 12 mg/m²/day by iv push on days 1,3,5 (application in patients > 65 years at days 1 + 3 only), etoposide 100 mg/m²/day by 1-hour iv infusion on days 1,2,3 (application in patients > 65 years at days 1 + 3 only), *PRIOR* azacitidine 100 mg/m²/day by subcutaneous injection on days −5 to day −1, idarubicin and etoposide as in STANDARD, *CONCURRENT* azacitidine 100 mg/m² per day by sc injection on days 1–5, idarubicin and etoposide as in STANDARD, *AFTER* Azacitidine 100 mg/m²/day by sc injection days 4–8, idarubicin and etoposide as in STANDARD, *ECOG* Eastern Cooperative Oncology Group, *WBC* white blood cells, *s-AML* AML after previous myelodysplastic syndrome or myeloproliferative neoplasm, *t-AML* therapy-related AML, *CEBPA*, CCAAT/enhancer-binding protein alpha, *DNMT3A* DNA methyltransferase 3A, *ASXL1* additional sex combs like 1, *RUNX1* runt-related transcription factor 1, *IDH* Isocitrate dehydrogenase, *TP53* tumor protein P53

^a^In case of bone marrow blasts < 20%, diagnosis of AML was established based on extramedullary disease or peripheral blood blasts > 20%

^b^patients can be listed in more than one of these overlapping categories

### Induction therapy

Of 104 patients treated during the first stage of the study, 49 (47%) achieved CR/CRi, 49 (47%) had refractory disease (RD), and 6 (6%) died. The number of patients achieving CR/CRi in the treatment arms PRIOR and CONCURRENT were 11 and 10, respectively. Therefore, both arms were stopped with the effective date 16 September 2011. The treatment arms STANDARD and AFTER were continued based on 14 patients achieving CR/CRi each. After recruitment of 168 patients in treatment arms STANDARD and AFTER, 45/84 (54%) and 37/84 (44%) patients achieved CR/CRi, respectively. Thus, only the STANDARD arm of the study was identified as effective according to the predefined criteria.

Overall, 268 patients received induction therapy, 126 (47%) patients achieved CR/CRi, 130 (49%) had RD, and 12 (4%) died during induction therapy. When salvage therapy outside the protocol was taken into account, CR/CRi was achieved in 161 patients (60%), 93 patients had RD (35%), and 14 died (5%), with all treatment arms showing similar increases in response.

A logistic regression model revealed biallelic *CEBPA* mutation as favorable (Odds Ratio [OR], 7.35; 95%-Confidence Interval [CI], 1.43–27.3), and adverse risk according to 2010 ELN risk classification as unfavorable (OR, 0.48; 95%-CI, 0.26–0.87) parameters for CR/CRi achievement. Within the final model, the estimates for the treatment arms containing azacytidine compared to STANDARD were as follows (PRIOR; OR, 0.59; 95%-CI, 0.25–1.37; CONCURRENT, OR, 0.44; 95%-CI, 0.19-1.05; AFTER, OR, 0.71; 95%-CI, 0.39-1.30).

We also explored the impact of genetics as predictive factor for the treatment effect on response. Patients with *IDH2*-mutated AML had a higher CR/CRi rate with STANDARD than with azacitidine-containing regimens (8/16 [50%] and 1/16 [6%], *p* = 0.02, respectively); similarly, CR/CRi rate in patients with *RUNX1*-mutated AML was in trend superior in STANDARD (10/15, 66%) compared to azacitidine-regimens (11/31 35%, *p* = 0.06). Twenty-seven patients had AML with *TP53* mutation, 13 patients were treated in AFTER and achieved a CR/CRi rate of 46%, whereas CR/CRi rate in the remaining patients was only 21% (*p* = 0.23). In patients with monosomal, complex (≥3 aberrations), or myelodysplasia-related karyotypes there was no difference in CR/CRi rates between STANDARD compared to all other arms (*p* = 0.66, *p* = 0.99, *p* = 0.46, respectively).

No differences in adverse events were observed in the four treatment arms except laboratory abnormalities all grades being more frequent in STANDARD and CONCURRENT as well as vascular abnormalities all grades and grade ≥ 3 predominantly observed in PRIOR (Table 2).

**Table 2:** Adverse Event occurring in first induction therapy according to treatment arm and CTCAE category

	STANDARD (n=100)<grade 3	≥grade 3	Prior (n=35)<grade 3	≥grade 3	CONCURRENT (n=34)<grade 3	≥grade 3	AFTER (n=99)<grade 3	≥grade 3	p-value ‘all grades *≥grade 3
Allergy/Immunology	13 (13)	1 (1)*	5 (14)	1 (3)*	3 (9)	0 (0)*	15 (15)	1 (1)*	0.75’ 0.69*
Cardiac Arrhythmia	3 (3)	3 (3)	1 (3)	1 (3)	3 (9)	2 (6)	6 (6)	5 (5)	0.33’ 0.81*
Cardiac General	14 (14)	9 (9)	7 (20)	2 (6)	6 (18)	2 (6)	9 (9)	8 (8)	0.61’ 0.97*
Coagulation	9 (9)	1 (1)	3 (9)	0 (0)	4 (12)	0 (0)	9 (9)	1 (1)	0.97’ 0.99*
Constitutional Symptoms	53 (53)	7 (7)	16 (46)	1 (3)	14 (41)	2 (6)	49 (49)	3 (3)	0.46’ 0.61*
Dermatology/Skin	42 (42)	3 (3)	10 (29)	1 (3)	16 (47)	0 (0)	33 (33)	7 (7)	0.49’ 0.31*
Endocrine	4 (4)	0 (0)	0 (0)	0 (0)	0 (0)	2 (6)	4 (4)	0 (0)	0.66’ 0.02*
Gastrointestinal	58 (58)	23 (23)	24 (69)	5 (14)	24 (71)	4 (12)	60 (61)	20 (20)	0.99’ 0.48*
Hemorrhage/Bleeding	25 (25)	6 (6)	6 (17)	2 (6)	9 (26)	3 (9)	28 (28)	5 (5)	0.66’ 0.85*
Hepatobiliary/Pancreas	0 (0)	2 (2)	1 (3)	0 (0)	0 (0)	1 (3)	0 (0)	2 (2)	0.99’ 0.92*
Infection	5 (5)	75 (75)	6 (17)	23 (66)	4 (12)	24 (71)	17 (17)	64 (65)	0.99’ 0.42*
Lymphatics	3 (3)	0 (0)	0 (0)	0 (0)	1 (3)	0 (0)	5 (5)	0 (0)	0.68’ 0.99*
Metabolic/Laboratory	45 (45)	16 (16)	8 (23)	5 (14)	14 (41)	5 (15)	25 (25)	14 (14)	0.007 ’0.99*
Musculoskeletal/Soft Tissue	2 (2)	4 (4)	3 (9)	0 (0)	0 (0)	0 (0)	8 (8)	2 (2)	0.22’ 0.48*
Neurology	27 (27)	1 (1)	10 (29)	1 (3)	9 (26)	2 (6)	28 (28)	1 (1)	0.95’ 0.22*
Ocular/Visual	4 (4)	1 (1)	1 (3)	0 (0)	3 (9)	0 (0)	8 (8)	1 (1)	0.52’ 0.99*
Pain	50 (50)	6 (6)	18 (51)	2 (6)	17 (50)	2 (6)	48 (48)	5 (5)	0.98’ 0.99*
Pulmonary/Upper Respiratory	20 (20)	5 (5)	4 (11)	6 (17)	7 (21)	3 (9)	24 (24)	6 (6)	0.80’ 0.12*
Renal/Genitourinary	43 (43)	2 (2)	7 (20)	1 (3)	13 (38)	3 (9)	31 (31)	5 (5)	0.09’ 0.25*
Sexual/Reproductive Function	1 (1)	0 (0)	1 (3)	0 (0)	0 (0)	0 (0)	0 (0)	0 (0)	0.45’ 0.99*
Syndromes	2 (2)	0 (0)	0 (0)	1 (3)	0 (0)	1 (3)	1 (1)	1 (1)	0.99’ 0.13*

*CTCAE* common terminology criteria for adverse Events, *STANDARD* cytarabine 100 mg/m²/day by continuous iv infusion on days 1–7, idarubicin 12 mg/m²/day by iv push on days 1,3,5 (application in older patients ( > 65 years) at days 1 + 3 only), etoposide 100 mg/m²/day by 1-hour iv infusion on days 1,2,3 (application in older patients at days 1 + 3 only), *PRIOR* azacitidine 100 mg/m²/day by subcutaneous injection on days −5 to day −1, idarubicin and etoposide as in STANDARD, *CONCURRENT* azacitidine 100 mg/m²/day by sc injection on days 1–5 before idarubicin and etoposide, Idarubicin and etoposide as in STANDARD, *AFTER* azacitidine 100 mg/m²/day by sc injection days 4–8, idarubicin and etoposide as in STANDARD

#### Consolidation therapy

Consolidation with HiDAC was administered for one cycle in 61 patients, for two cycles in 45 patients, and for three cycles in 37 patients. Within the protocol, 49 patients proceeded to allogeneic HCT in first CR/CRi; overall, 88 patients received allogeneic HCT in first CR/CRi, 45 patients with RD, and 21 patients after relapse. Forty-six patient received a matched-related donor transplant, 107 a matched-unrelated, and one patient a transplant from a haploidentical donor.

#### Maintenance therapy

Maintenance therapy with azacitidine was started in 15 patients. Median number of applied cycles was 5 (range, 1–24), with 2 patients receiving the intended 24 cycles. In 13 patients maintenance was terminated early (relapse, *n* = 12; toxicity, *n* = 1).

#### Survival analysis

**Fig. 2:**
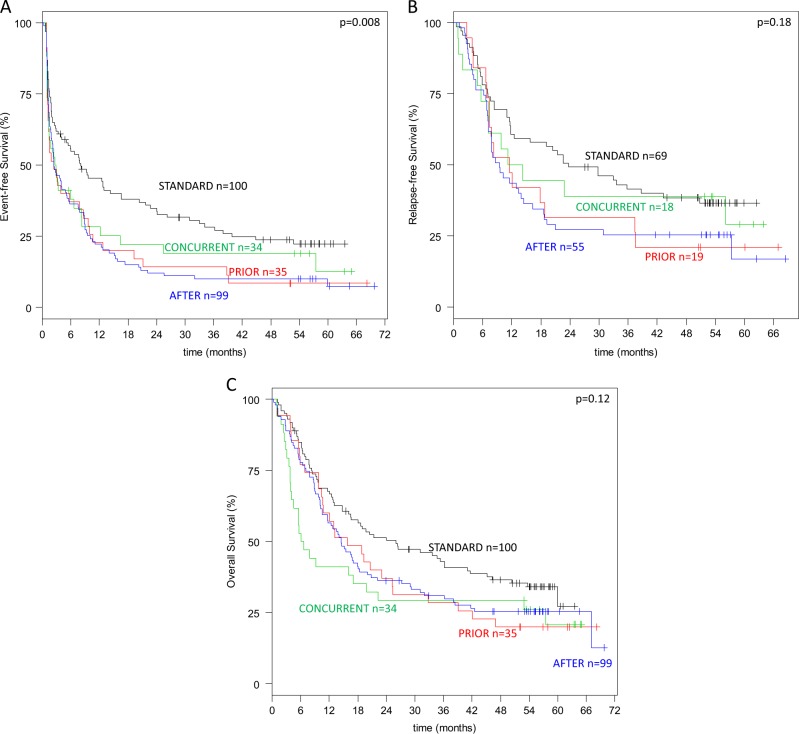
Kaplan-Meier plots illustrating the influence of upfront randomization on event-free (EFS) (**a**), relapse-free (RFS) (**b**), and overall survival (OS) (**c**)

Median follow-up was 56 months (95%-CI, 54–57 months). Overall median and 4-year EFS, RFS, and OS were 3.5 months, 15 months, 16 months, and 16% (95%-CI, 12–21%), 30% (95%-CI, 23–39 months), 29% (95%-CI, 24–35%), respectively. EFS (Fig. 2a) was significantly different among the four arms (*p* = 0.008), with inferior EFS in all three azacitidine arms compared to STANDARD (p < 0.001). RFS and OS (Fig. 2b, c) were not significantly different among the four study arms (*p* = 0.18, *p* = 0.12; respectively), but inferior when the three azacitidine arms were compared to STANDARD (*p* = 0.04, *p* = 0.03; respectively). Even in patients proceeding to an allogeneic HCT in first CR/CRi, RFS was in trend inferior in the three azacitidine arms compared to STANDARD (*p* = 0.07). In an Anderson Gill regression model including allogeneic HCT performed in first CR/CRi as a time-dependent covariable, all azacitidine arms showed worse outcome; further unfavorable factors were higher age, male sex, presence of a *TP-53* mutation, and ELN high-risk. Favorable factors were biallelic *CEBPA* mutations, female gender, and allogeneic HCT in first CR/CRi (Table 3).

**Table 3:** Anderson-Gill regression model on the endpoint overall survival including allogeneic hematopoietic-cell transplantation performed in first CR/CRi as a time-dependent covariable

	HR	95%-CI	*p* value
Treatment Arm			
PRIOR	1.58	0.95–2.63	0.08
CONCURRENT	1.84	1.02–3.37	0.04
AFTER	1.49	1.00–2.23	0.05
Age (1 year difference)	1.02	1.01 1.03	0.009
Female sex	0.69	0.49 0.98	0.04
*CEBPA*-dm	0.48	0.23 1.01	0.06
*TP53*-mut	2.26	1.25 4.07	0.007
2010 ELN high-risk	1.67	1.10 2.53	0.02
Allogeneic HCT in first CR/CRi	0.36	0.22 0.57	<0.0001

*HCT* hematopoietic-cell transplantation, *CR* complete remission, *CRi* CR with incomplete blood count recovery, *STANDARD* cytarabine 100 mg/m²/day by continuous iv infusion on days 1–7, idarubicin 12 mg/m²/day by iv push on days 1,3,5 (application in patients > 65 years at days 1 + 3 only), etoposide 100 mg/m²/day by 1-hour iv infusion on days 1,2,3 (application in patients > 65 years at days 1 + 3 only), *PRIOR* azacitidine 100 mg/m²/day by subcutaneous injection on days −5 to day −1, idarubicin and etoposide as in STANDARD, *CONCURRENT* azacitidine 100 mg/m²/day by sc injection on days 1–5, idarubicin and etoposide as in STANDARD, *AFTER* azacitidine 100 mg/m²/day by sc injection on days 4–8, idarubicin and etoposide as in STANDARD, *CEBPA*-dm biallelic CCAAT/enhancer-binding protein alpha mutations, *TP53* tumor protein P53, *mut* mutation, *ELN* European LeukemiaNet.

## Discussion

Based on the improved understanding of the molecular pathogenesis of AML, new treatment strategies targeting specific molecular defects have been implemented within treatment trials of the German-Austrian AML Study Group (AMLSG), such as FLT3 inhibition in AML with *FLT3*-ITD (ClinicalTrials.gov Identifier: NCT01477606) [19], KIT-inhibition in CBF-AML (NCT00850382) [17], and the use of gemtuzumab ozogamicin in AML with *NPM1* mutations (NCT00893399, EudraCT 2009-011889-28) [18].

The remaining patients not eligible for these targeted approaches were mainly patients exhibiting an intermediate-2 or high-risk according to the 2010 ELN categorization [5]. Furthermore, 20% of patients had *RUNX1*-mutated AML, 15% *ASXL1*-mutated AML, and 13% *TP53*-mutated AML, all markers that are categorized within the adverse-risk group in the 2017 ELN risk stratification [34]; in addition, 25% of patients had *IDH1*/*IDH2*-mutated AML. Based on previous observations that hypomethylating agents may be particularly active in AML with poor-risk disease features, such as adverse-risk genetics, myelodysplasia-related changes, or specific gene mutations (e.g., *TP53*) [10–12, 35–38], our hypothesis was that these patients would benefit from incorporation of azacitidine within a regimen of intensive induction chemotherapy. We opted to substitute cytarabine by azacitidine within the commonly used ICE regimen based on share common chemical and biological characteristics as well as same metabolic pathways of incorporation into DNA. Since different sequences of azacitidine administration may affect efficacy, we employed three different investigational regimens, azacitidine given prior, concomitantly, and after chemotherapy.

On the basis of the optimal two-stage design of Simon, two arms of the study, PRIOR and CONCURRENT, had to be stopped early due to insufficient response rates. The study arm AFTER with azacitidine given after idarubicin and etoposide was similarly effective in the first stage than STANDARD, but in the second stage also failed with inferior induction results. Thus, all three investigational treatment arms were associated with an inferior response rate compared to STANDARD. Although comparable CR/CRi rates were achieved in all arms if high-dose cytarabine-based salvage therapy was included in the analysis, the inferior initial response in all azacitidine-containing arms translated into inferior EFS, RFS, and OS. Thus, the results of this study suggest that cytarabine remains an important component of induction therapy even in patients with adverse risk. Furthermore, our results are comparable to those reported by Müller-Tidow et al. adding azacitidine prior to intensive induction therapy [39]. In contrast to that study, we did not identify additive toxicity due to azacitidine, probable because of the omission of cytarabine in the azacitidine-containing treatment arms.

In exploratory analyses we looked at the impact of genetics on response to therapy. Of note, in patients exhibiting a complex, monosomal or myelodysplasia-related karyotype there was no beneficial effect of adding azacitidine to intensive induction therapy. Azacitidine was associated with significant inferior response rates in patients with *IDH2*- and *RUNX1*-mutated AML. In the AZA-AML-001 trial evaluating azacitidine versus conventional care regimens [10], mutations in two genes, that is *FLT3* and *TET2*, were shown to negatively impact OS within the azacitidine treatment arm [12]. Of the 27 patients with *TP53*-mutated AML in our trial, 13 patients were treated in AFTER and achieved a CR/CRi rate of 46%, whereas CR/CRi rate in the remaining patients was only 21%, but this difference was not statistically significant (*p* = 0.23). Activity of hypomethylating agents in patients with *TP53*-mutated AML has been demonstrated in two previous trials. In a study of decitabine in patients with AML or MDS, those with *TP53* mutations had a 100% response rate compared with a 41% response rate in patients with wild-type *TP53*, however responses were not durable [11]. In the AZA-AML-001 trial, median OS was prolonged by almost 5 months in patients with *TP53* mutations receiving azacitidine compared with patients receiving conventional care regimens [12].

In conclusion, in this study of patients with AML exhibiting predominantly higher risk disease features the substitution of cytarabine by azacitidine within an intensive chemotherapy regimen of idarubicin and etoposide failed to improve response rates. On the contrary, two investigational arms had to be stopped early, and all three investigational arms were associated with poorer outcome compared to the standard ICE regimen.
